# The 2018 Japan Floods Increased the Frequency of *Yokukansan* Prescriptions Among Elderly: A Retrospective Cohort Study

**DOI:** 10.3389/fnut.2021.777330

**Published:** 2022-01-24

**Authors:** Ryoko Ishida, Shuhei Yoshida, Saori Kashima, Yuji Okazaki, Masatoshi Matsumoto

**Affiliations:** ^1^Department of Community-Based Medical System, Graduate School of Biomedical and Health Sciences, Hiroshima University, Hiroshima, Japan; ^2^Environmental Health Sciences Laboratory, Graduate School of Advanced Science and Engineering, Hiroshima University, Hiroshima, Japan; ^3^Department of General Internal Medicine, Kitahiroshimacho Yahata Clinic, Hiroshima, Japan

**Keywords:** natural disaster, mental health care, Yokukansan, Kampo (traditional Japanese herbal medicine), elderly people, National Database of Health Insurance Claims, prescription, rural health services

## Abstract

**Objective:**

The impact of the 2018 Japan Floods on prescriptions of *Yokukansan* was evaluated.

**Methods:**

This was a retrospective cohort study based on the National Database of Health Insurance Claims which covers all the prescriptions issued in Japan. Participants were patients aged 65 or older who received any medical care at medical institutions located in the three most-severely affected prefectures between 1 year before and after the disaster. We analyzed the number of new prescriptions of *Yokukansan* and other Kampo drugs among those who had not been prescribed any Kampo for 1 year before the disaster. Kaplan-Meier analysis and a Cox proportional hazards model were used to evaluate the risk of the disaster for a new prescription.

**Results:**

Subjects comprised 1,372,417 people (including 12,787 victims, 0.93%). The hazard ratio (HR) of the disaster for *Yokukansan* prescriptions was 1.49 [95% confidence intervals (CI): 1.25–1.78], and 1.54 (95% CI: 1.29–1.84) in the crude and age-sex adjusted model, respectively. The HR of the disaster for prescription of other Kampo drugs in the crude and adjusted model was 1.33 (95% CI: 1.27–1.39), and 1.32 (95% CI: 1.27–1.38), respectively. The magnitude of increase of victims prescribed *Yokukansan* (31.4%) was statistically higher than for those prescribed other Kampo drugs (19.3%) (*p* < 0.001).

**Conclusion:**

The disaster increased prescriptions of both *Yokukansan* and other Kampo drugs among elderly victims. The increase was more remarkable in *Yokukansan* than other Kampo drugs. Clinicians and policymakers should be aware of the increased need for *Yokukansan* in times of natural disaster.

## Introduction

Global warming is causing rapid climate changes, and the scale of disasters is increasing worldwide. Therefore, it has become important around the world to investigate effective relief activities to cope with such disasters.

The 2018 Japan Floods, which occurred from June 28 to July 8, 2018, had a widespread impact across the country, especially in western Japan. The amount of financial damage was the second highest among water-related disasters in Japan. The number of deaths and missing persons was 271 (55.7% was elderly people over 65 years old) (1), which was the most since The July 1982 Torrential Rains in Nagasaki (2, 3). Total damaged houses were 29,473 including completely destroyed, half-destroyed, partially destroyed, and flooded above the floor level (1), and the total damage amounted to ~1.41 trillion yen/12.66 billion dollars (1 dollar = 111.37 yen, converted at Central rate, average in the month, Tokyo Market as of July 2018) (4). The damage was concentrated in Okayama, Hiroshima, and Ehime prefectures; with 90.8% of deaths and missing persons, 84.9% of total damaged houses (1), and 66.1% of the total amount of damage (4) occurring in these areas.

In this study, among the numerous health problems caused by the torrential rains (5), we examined the use of the Kampo medicines for the mental symptoms of the elderly, who make up the majority of the disaster victims. It is well known that various mental disorders, for example, depression, anxiety, and post-traumatic stress disorder (PTSD), increase due to natural disasters (6). Anti-anxiety and hypnotic drugs used for such cases are associated with risks such as lightheadedness and falling, and benzodiazepines may cause dependency. Kampo medicines do not have such side effects and are safer to use, so they are used in combination with Western medicines or when Western medicines are being tapered or discontinued. In Japan, 148 Kampo formulas are covered by the public health insurance and are frequently prescribed for female and elderly persons by more than 80% of medical doctors. Kampo is useful when a disaster occurs and medical equipment cannot be used, because it is prescribed based on symptoms, inquiry, and findings. Indeed, Kampo therapy was provided in evacuation centers after the Great East Japan Earthquake and showed beneficial effects (7). It is expected that the need for Kampo therapy will rise in response to the remarkable increase in the mental problems caused by a natural disaster, but there has been no research to confirm this.

Out of 148 Kampo drugs, *Yokukansan* (YKS) is well known Kampo formula in Japan that is used to treat mental symptoms. It consists of seven kinds of medical herbs and its indications are for weakness, nervousness, insomnia, crying at night in children, and childhood neurosis. Indicated symptoms may also include agitation and irritability. YKS is listed in guidelines by the Japan Geriatrics Society as a Kampo that has been validated for efficacy and safety in the elderly (8, 9). Moreover, it has been reported in randomized controlled trials (RCTs) (10, 11) and meta-analysis (12) that YKS is effective in relieving agitation symptoms related to behavioral and psychological symptoms of dementia (BPSD), such as hallucinations, delusions, agitation, and violence. Therefore, in the guideline for dementia, it is written that atypical antipsychotics such as risperidone and aripiprazole are effective in treating agitation, and the use of YKS may also be considered (13). In addition, YKS is selected out of a number of Kampo drugs to be carried as a medicine by the Japan Medical Association Team (JMAT), which is managed by the Japan Medical Association to support victims in disaster-affected areas. Therefore, it is possible that mental health problems which could be treated by YKS arise from a natural disaster, and that YKS prescriptions increase in disaster-affected areas to improve the psychiatric condition of elderly persons.

In this study, we quantitate the usage of YKS to care the mental symptoms of the elderly persons who were affected by the disaster, by using the probability of new prescriptions of YKS as an indicator. We also clarify if the magnitude of increase of persons prescribed YKS is greater than that of other persons prescribed other 147 Kampo drugs.

## Methods

### Research Design

Retrospective cohort study.

### Data Source

This study is based on the NDB managed by the Ministry of Health, Labor and Welfare (permission no. 0710-4). The NDB is one of several government-maintained nationwide healthcare-related databases in Japan. All residents of Japan are covered by the public health insurance, and thus the database includes data on all the drugs prescribed in Japan.

### Settings

Okayama, Hiroshima, and Ehime prefectures were selected as the target areas for the location of medical institutions, where the damage was concentrated as described in the introduction. The survey period was set between July 2017 and June 2019, which is a period of 1 year before the disaster and 1 year after the disaster. The subjects of this study were those who were 65 years old or older and visited and were issued health insurance claims from medical institutions located in Okayama, Hiroshima, and Ehime prefectures among the registrants in the database. Among the subjects, those who were identified as disaster-victims by the local government were also considered as disaster-victims in this study. The rest of the participants were identified as non-victims and analyzed separately.

### Definition of Disaster-Victims

During the 2018 Japan Floods, it was announced by the Japanese government that medical insurance co-payments would be fully exempted for victims (14). We therefore defined a “disaster-victim” as a person who was listed as a disaster-victim in the special notes on health insurance claims issued after the disaster. However, people whose medical expenses had been already funded by the government, such as livelihood recipients and atomic bomb survivors who were provided with an atomic bomb survivor's certificate, were not eligible for exemption due to the disaster, therefore were categorized as non-victims even if they were affected.

Government-certified victims were fully exempted from the general out-of-pocket charge (10–30% of the total medical expense) for any medical services and were enrolled as such in the NDB. A disaster-victim was certified by the local government in their residential municipality. The criteria for a certified “disaster-victim” fell into one of the following categories: (1) residential house was completely or partially destroyed, burned down, flooded above the floor level, or similarly damaged, (2) family member who had financially supported the person was killed or suffered severely, or was missing.

### Targeted Kampo Drugs

Regardless of whether a participant was affected by the disaster or not, YKS was the fourth most commonly prescribed Kampo drug both before and after the disaster (4.9–5.3%) (Supplementary Figures 1A,B), and it was the most commonly prescribed antipsychotic Kampo drug (27.3–28.6%) (Supplementary Figures 1C,D). Moreover, among 39 Kampo drugs approved by the Japan's Ministry of Health, Labor and Welfare to improve psychological symptoms, YKS is the only Kampo drug whose effectiveness is supported with sound scientific evidence (15, 16). For these reasons, we chose YKS as the subject of our analysis.

### Variables

Micro analysis: The month when the new prescription of YKS was issued for the subjects was identified within the period of the study. Similarly, the month when the new prescription of other Kampo drugs was issued for the subjects was identified. Data on whether the subject was affected by the disaster, age, and gender were also extracted. However, those who had received prescriptions of any Kampo drugs before the disaster were excluded. For age categories and gender, we adopted the numbers on the first health insurance claims within the period of the study.

Aggregate analysis: In the subjects, we counted the number of people who received a prescription of YKS during the period of the study for 1 year before and after the disaster respectively. Likewise, the number of people who received prescriptions of any of 147 Kampo drugs other than YKS was also counted.

### Statistical Analysis

Using micro analysis, we quantitatively examined the extent to which the disaster increased the incidence of new prescriptions of YKS and other Kampo drugs. Aggregate analysis was used to compare the change in the magnitude of increase in the number of people prescribed between YKS and other Kampo drugs before and after the disaster. The magnitude of increase was calculated for all subjects for 1 year before and 1 year after the disaster. The difference was examined by a proportion test.

#### Descriptive Statistics

We described the basic characteristics represented by discrete variables for victims and non-victims and conducted a χ-square test to examine the differences in the basic attributes used in the micro analysis, such as age and gender, between disaster victims and non-victims.

#### Examination of the Incidence of New Prescriptions Using Kaplan-Meier Failure Estimates

The new incidence rate of YKS prescriptions verses that of other Kampo drugs were described graphically with the Kaplan-Meier analysis. The start point of the observation was July 2018 and prescriptions were recorded on a monthly basis. The rate was compared between the two groups with the log-rank test.

#### Examination of the Impact of the Disaster on the Event Probability of New Prescriptions Using the Cox Proportional Hazards Model

We used the Cox proportional hazards model to quantitatively measure the impact of the disaster on the event probability of new prescriptions of YKS and the other Kampo drugs, respectively. The period of observation was for 1 year following July 2018. In addition to the crude model, in which the effect of the disaster was the only variate, multivariate estimation which adjusted for gender and age category was also conducted (adjusted model). After examination of the Cox proportional hazards model, we confirmed that proportional hazard assumption was clear.

We performed all statistical analyses using STATA/MP version 16 (Stata Corp, 2019).

### Ethics

The need for informed consent was obviated by the anonymity of the NDB. This study was permitted by the institutional review board of Hiroshima University (No. E-1688).

## Results

### Micro Analysis

The subjects were extracted by restricting the sample to those who had not received any prescriptions of the 148 Kampo drugs in 1 year prior to the disaster. The demographics of those subjects are presented in Table 1. The total number of participants aged 65 years old or more was 1,372,417, and 0.93% were disaster-victims (12,787 people). Participants in their seventies were relatively more affected compared to the other age categories (*p* < 0.001).

**Table 1:** Basic characteristics of study subjects.

**Participants**		**Victims**	**Non-victims**	**p-value**
		**12,787 (0.93)**	**1,359,630 (99.07)**	
Age classification, *n* (%)	65–69	3,358 (26.26)	373,167 (27.45)	*p* < 0.001
	70–74	3,168 (24.78)	311,035 (22.88)	
	75–79	2,440 (19.08)	237,544 (17.47)	
	80-	3,821 (29.88)	437,884 (32.21)	
Gender, *n* (%)	Male	5,649 (44.18)	602,001 (44.28)	*p* = 0.822
	Female	7,138 (55.82)	757,629 (55.72)	
Prefecture, *n* (%)	Okayama	5,876 (45.95)	407,601 (29.98)	*p* < 0.001
	Hiroshima	3,579 (27.99)	566,009 (41.63)	
	Ehime	2,700 (21.12)	316,596 (23.29)	
	Missing or other	632 (4.94)	69,424 (5.11)	
Incidence of new prescription of *Yokukansan*	Prescribed participants	125 (0.98)	8,913 (0.66)	*p* < 0.001
after disaster, *n* (%)	Non-prescribed participants	12,662 (99.02)	1,350,717 (99.34)	
Incidence of new prescription of other Kampo	Prescribed participants	1,981 (15.49)	161,935 (11.91)	*p* < 0.001
drugs after disaster, *n* (%)	Non-prescribed participants	10,806 (84.51)	1,197,695 (88.09)	

The percentage of all participants by gender was 44.28% male and 55.72% female, which was similar to the percentage of males and females of the same age categories who lived in Okayama, Hiroshima, and Ehime prefectures (42.71 and 57.29%, respectively) as of January 2018 (17). A gender difference in the degree of disaster vulnerability was not observed (*p* = 0. 822).

Of the three prefectures, Okayama prefecture was the most common location of the medical institutions visited by victims, accounting for 45.95%. The test results showed that the probability of being affected by the disaster was significantly higher for participants who visited medical institutions in Okayama prefecture compared to the other two prefectures (*p* < 0.001).

The number of victims newly prescribed for YKS amounted to 125 (0.98% of all victims), while non-victims amounted to 8,913 (0.66% of all non-victims). The proportion of new prescriptions of YKS for the disaster-victims was significantly higher than for non-victims (*p* < 0.001).

The number of disaster-victims prescribed for other Kampo drugs amounted to 1,981 (15.49% of all victims), while non-victims amounted to 161,935 (11.91% of all non-victims). The proportion of new prescriptions of other Kampo drugs for victims was significantly higher than for non-victims (*p* < 0.001).

**Figure 1:**
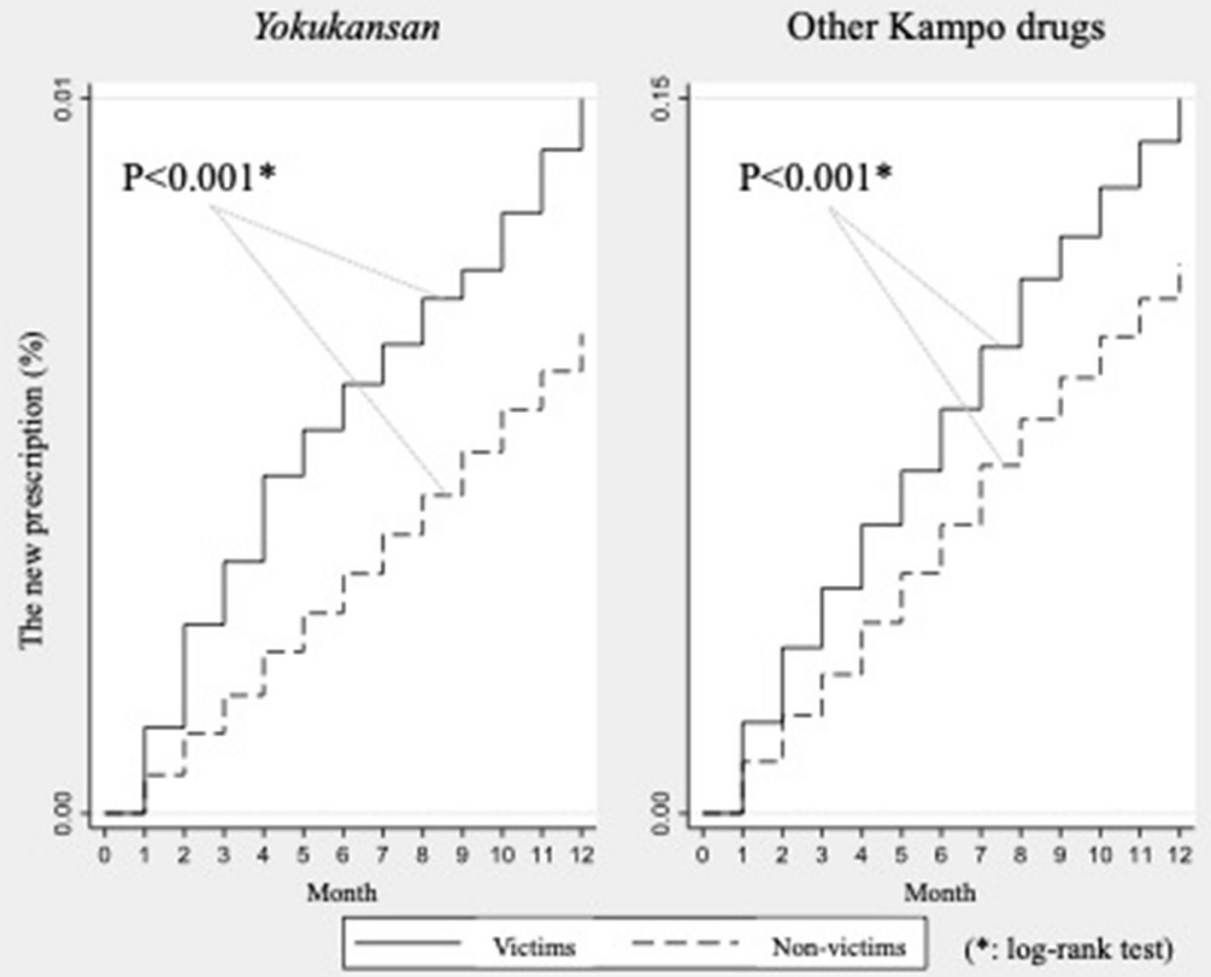
Kaplan-Meier failure curves for the patients of newly prescribed *Yokukansan* and other Kampo drugs.

Kaplan-Meier failure curves for the patients newly prescribed YKS and other Kampo drugs are shown in Figure 1. As a result of a series of log-rank tests, new prescriptions of YKS (Figure 1, left panel) occurred at a higher rate in disaster-victims compared with non-victims (*p* < 0.001) and was concentrated in the 2 months after the disaster. We also estimated new prescriptions of other Kampo drugs using the same method (Figure 1, right panel). New prescriptions were observed more frequently among victims than non-victims, which was similar to the results for YKS (*p* < 0.001). In the contrast to YKS, however, a concentration of new prescriptions for other Kampo drugs at a specific time point was not observed.

Table 2A shows the results of an examination of new prescriptions for YKS using a Cox proportional hazards model. The hazard ratio (HR) for the disaster in the crude model was 1.49 (95% confidence intervals (CI): 1.25–1.78), while that of the adjusted model adjusted by age categories and gender was 1.54 (95% CI: 1.29–1.84), which indicated that the probability of new prescriptions of YKS had increased due to the disaster. We also examined the results by age categories. HR for the age group 70–74 was 1.90 (95% CI: 1.72–2.10), age group 75–79 was 3.57 (95% CI: 3.25–3.92), and age group more than 80 was 8.35 (95% CI: 7.69–9.08), which indicated that YKS was more likely to be prescribed for higher age categories. On the other hand, HR for the disaster in the crude model for other Kampo drugs was 1.33 (95% CI: 1.27–1.39), where the adjusted model was 1.32 (95% CI: 1.27–1.38), both of which were >1 as well as for YKS (Table 2B). Although the 95% CI for YKS and other Kampo drugs slightly overlapped, HR for YKS tended to be greater than the other Kampo drugs.

**Table 2:** Hazard ratios and 95% confidence intervals for a new prescription **(A)** *Yokukansan*, **(B)** Other Kampo drugs.

		**Hazard ratio (95% CI)**	
		**Crude**	**Adjusted**
**(A)**			
Victims (Ref = Non-victims)		1.49 (1.25–1.78)	1.54 (1.29–1.84)
Age categories (Ref = 65–69)	70–74		1.90 (1.72–2.10)
	75–79		3.57 (3.25–3.92)
	80–		8.35 (7.69–9.08)
Gender (Ref = male)			1.07 (1.03–1.12)
**(B)**			
Victims (Ref = Non-victims)		1.33 (1.27–1.39)	1.32 (1.27–1.38)
Age categories (Ref = 65–69)	70–74		1.12 (1.11–1.14)
	75–79		1.26 (1.25–1.28)
	80–		1.03 (1.02–1.04)
Gender (Ref = male)			1.15 (1.14–1.16)

### Aggregate Analysis

When the target population was expanded to include all people regardless of whether or not they had been prescribed Kampo before the disaster, the total number was 1,812,373, of which 16,396 were victims (Table 3). The number of patients who were prescribed YKS before and after the disaster was 226 and 297, respectively, and the magnitude of increase (31.4%) was significantly greater than that of other Kampo drugs (19.3%) (*p* < 0.001). In other words, the impact of the disaster on prescriptions was greater for YKS.

**Table 3:** Magnitude of increase in the number of subjects prescribed *Yokukansan* and other Kampo Drugs from before to after the disaster.

	**From July 2017 to June 2018**	**From July 2017 to June 2018**	MI^*^	***p* value**
	**(A)**	**(B)**	**(B-A)/(A)**	
	***n***	***n***	**%**	
**Victims**				
*Yokukansan*	226	297	31.4	<0.001^**^
Other Kampo drugs	3,448	4,113	19.3	
**Non-victims**				
*Yokukansan*	30,269	30,226	−0.1	<0.001^**^
Other Kampo drugs	416,431	412,353	−1.0	

* *Magnitude of increase*.

** *Proportion test compared the difference between MI of Yokukansan and other Kampo drugs*.

## Discussion

### Main Findings

The 2018 Japan Floods increased prescriptions of both YKS and other Kampo drugs among elderly victims. The degree of increase in opportunities for prescribing YKS was greater than that of other Kampo drugs. Supported by the hazard model estimation adjusted for age and gender, these results were confirmed to be robust. The new prescriptions of YKS in victims were most frequently observed 2 months after the disaster. And YKS was more likely to be prescribed for higher age categories. These two characteristics were not observed in other Kampo drugs. Both YKS and other Kampo drugs were more likely to be prescribed for females, but YKS tended to be prescribed more often regardless of gender. To the best of our understanding, this is the first cohort study to quantitatively examine the impact of natural disasters on the prescriptions of Kampo.

### Considerations

The locations of the medical institutions where the affected study subjects visited were, in order of the number of subjects, Okayama, Hiroshima, and Ehime prefectures. This ranking was the same as the ranking of the amount of damage: 421 billion yen/3.89 billion dollars for Okayama, 339 billion yen/3.13 billion dollars for Hiroshima, and 170 billion yen/1.57 billion dollars for Ehime prefecture.

“*Yokukansan*” means to adjust the function of “*kan*,” meaning “liver” in Japanese. However, in terms of Kampo medicine, “liver” does not mean “liver” as described in Western medicine. The naming indicates a concept that includes various functions such as emotion, autonomic nervous function, function of the eyes and muscles, and regulation of blood distribution. In other words, YKS has the effect of suppressing mental tension, especially persistent anger caused by dysfunction of the “liver,” hence its name (18).

In times of disaster, the Kampo that are prescribed for psychiatric symptoms as represented by YKS are safe and convenient to use when persons are dealing with an unusual and unfamiliar living environment, such as an evacuation center, because they do not have side effects such as undifferentiated dizziness as often experienced with antipsychotics. However, it is noted that although it contains a relatively small amount of glycyrrhiza (1.5 g/day), it is important to pay attention to its possible side effect of pseudohyperaldosteronism (19).

#### Post-disaster Mental Health Care and Yokukansan

Our study clarified that the the probability of new prescriptions of all Kampo statistically increased after the disaster. This was the case in particular for YKS, which is effective for mental symptoms such as irritation, and was more likely to be prescribed than other Kampo drugs for older patients. YKS is a commonly prescribed Kampo in general and more so it was in times of natural disaster.

Several possibilities can be raised regarding the cause of the high probability of prescriptions for YKS. First, YKS might be used for mental symptoms associated with cognitive decline such as for the observed correlation between damage to houses and cognitive decline among elderly people in the 2011 Great East Japan Earthquake (20). Next, considering the psychological and emotional aspects of the aftermath of the disaster period, well-known mental health problems include posttraumatic stress disorder (PTSD), depression, anxiety, and substance use disorder (21), and studies on anger are still limited. However, it has been reported that about 10% of the people who were highly affected by bushfire in Australia felt three times as much anger as those who were less affected (22). The results of analysis using the same sample showed that the possibilities for an increase in PTSD, depression, severe mental illness (SMI) in the aftermath of a bushfire was 19, 11, and 6%, respectively (23), which indicates that “anger” should be more considered as an important issue among these mental disorders. Our results indicate that YKS was more likely to be prescribed confirms that care for anger is necessary after a disaster.

#### The Mechanism of Action for Yokukansan

The neuromodulators of mental states such as anger and violence include serotonin and glutamate, along with dopamine, norepinephrine, GABA, and acetylcholine (24). There are a number of research studies on the mechanism of action of YKS involving the serotonin nervous system and the glutamate nervous system to adjust emotion. For the serotonin nervous system, pharmacological effects are exerted by acting on its receptors (25–27), and for the glutamatergic nervous system, by consequently reducing the neurotoxicity of glutamate (28–31).

#### Disasters and Kampo Therapy

Kampo therapy in the aftermath of large-scale disasters has been practiced in the past, mainly in Japan and China. According to a review of 12 papers on this topic, Kampo therapy has been used for primary care conditions such as common cold, constipation, insomnia, and for other conditions, for instance, irritation, PTSD, trauma, dizziness, and pain (32). It is difficult to generalize about the psychosomatic symptoms that may occur after a disaster because of differences in the season in which a disaster occurred or duration at evacuation shelters. Nevertheless, the symptoms mentioned above are considered to be medically unexplained physical symptom (MUPS), such as headache, fatigue, diarrhea, abdominal pain, and palpitations, which have been reported to persist for years after a disaster (33). Since Kampo therapy is well adapted to the treatment of MUPS, it is thought that new prescriptions increased across Kampo.

In addition, many studies show that the care needed after a disaster changes depending on the period. For example, the treatment of trauma and primary care were needed in first 2 months, while the treatment for PTSD started 6–8 months later after the disaster (32). In our study, new prescriptions of YKS were most frequent 2 months after the disaster. This observation was consistent with a previous report where the prescription sequence of YKS was eight during the first 2–4 weeks after the Great East Japan Earthquake, but second during the first 1–2 months after the earthquake among Kampo formulas prescribed (7).

#### Health Economics Perspective

It is also important to consider the possibility that the exemption of co-payments for medical expenses for victims might have increased the amount of drug prescriptions in general. The price elasticity of outpatient care under the medical care system for the elderly in Japan has been reported to be −0.125 to −0.076 (34). When the health insurance co-payment rate for each age group was applied to the target group of victims in this study, the average co-payment rate, calculated from the age distribution, was 18.02%. Assuming that the co-payment was totally exempted, the increase of care demand was calculated to stay at 1.37 to 2.25%. Thus, the increase in demand for medical care due to the co-payment exemption would be minimal, and the substantial increase in prescriptions of YKS and other Kampo drugs could not be explained only by price elasticity. In addition, the opportunities for prescribing YKS increased more than that for other Kampo drugs, and this difference was independent of price elasticity, suggesting that a medical condition had arisen that led to an indication for YKS in the victims.

### Strengths

This study is the first to describe a change in prescriptions of YKS using completely covered data, such as health insurance claims. Japan has a universal health insurance system, which covers almost all medical practices for all residents in the country. Therefore, we were able to quantitatively measure the impact of the disaster in a manner that was close to completely covered for those that had been prescribed YKS at the doctor's discretion. In addition, the data is highly accurate because it is based on a database managed by the government.

### Remaining Issues

Our study has several limitations. We were not able to examine the symptoms of individual patients and efficacy of YKS. Furthermore, medical institutions were not categorized in terms of size, department, whether or not the doctors were Kampo specialists, the content of the medical services, and accessibility. Lastly, in this study, the victims were identified based on the data from health insurance claims. However, it is presumed that there were a certain number of people who were affected, but had not applied for certification as a disaster-victim. In other words, the non-victims in this study might have included people who were affected by the disaster, and the results obtained from this study may be lower than the actual estimates.

## Conclusion

The number of prescriptions of both YKS and other Kampo drugs increased among elderly persons affected by a natural disaster. The increase was more remarkable for YKS than other Kampo drugs. Clinicians and policy makers should be aware of the increased need for Kampo drugs during natural disasters and need to establish a stable and smooth supply system of Kampo during normal periods to meet such needs.

## Data Availability Statement

The original contributions presented in the study are included in the article/Supplementary Material, further inquiries can be directed to the corresponding author/s.

## Ethics Statement

The studies involving human participants were reviewed and approved by Hiroshima University. Written informed consent for participation was not required for this study in accordance with the national legislation and the institutional requirements.

## Author Contributions

RI, SY, and MM were involved in writing of the manuscript, contributed to conception, and design of the study. SY performed the statistical analysis. All authors contributed to manuscript revision, read, and approved the submitted version.

## Conflict of Interest

The authors declare that the research was conducted in the absence of any commercial or financial relationships that could be construed as a potential conflict of interest.

## Publisher's Note

All claims expressed in this article are solely those of the authors and do not necessarily represent those of their affiliated organizations, or those of the publisher, the editors and the reviewers. Any product that may be evaluated in this article, or claim that may be made by its manufacturer, is not guaranteed or endorsed by the publisher.
