# Supplementary figures and images for: The 2018 Japan Floods Increased the Frequency of Yokukansan Prescriptions Among Elderly: A Retrospective Cohort Study
Source: Front Nutr. 2022 Jan 24;8:777330. doi: 10.3389/fnut.2021.777330 (PMC8819099; doi:10.3389/fnut.2021.777330)

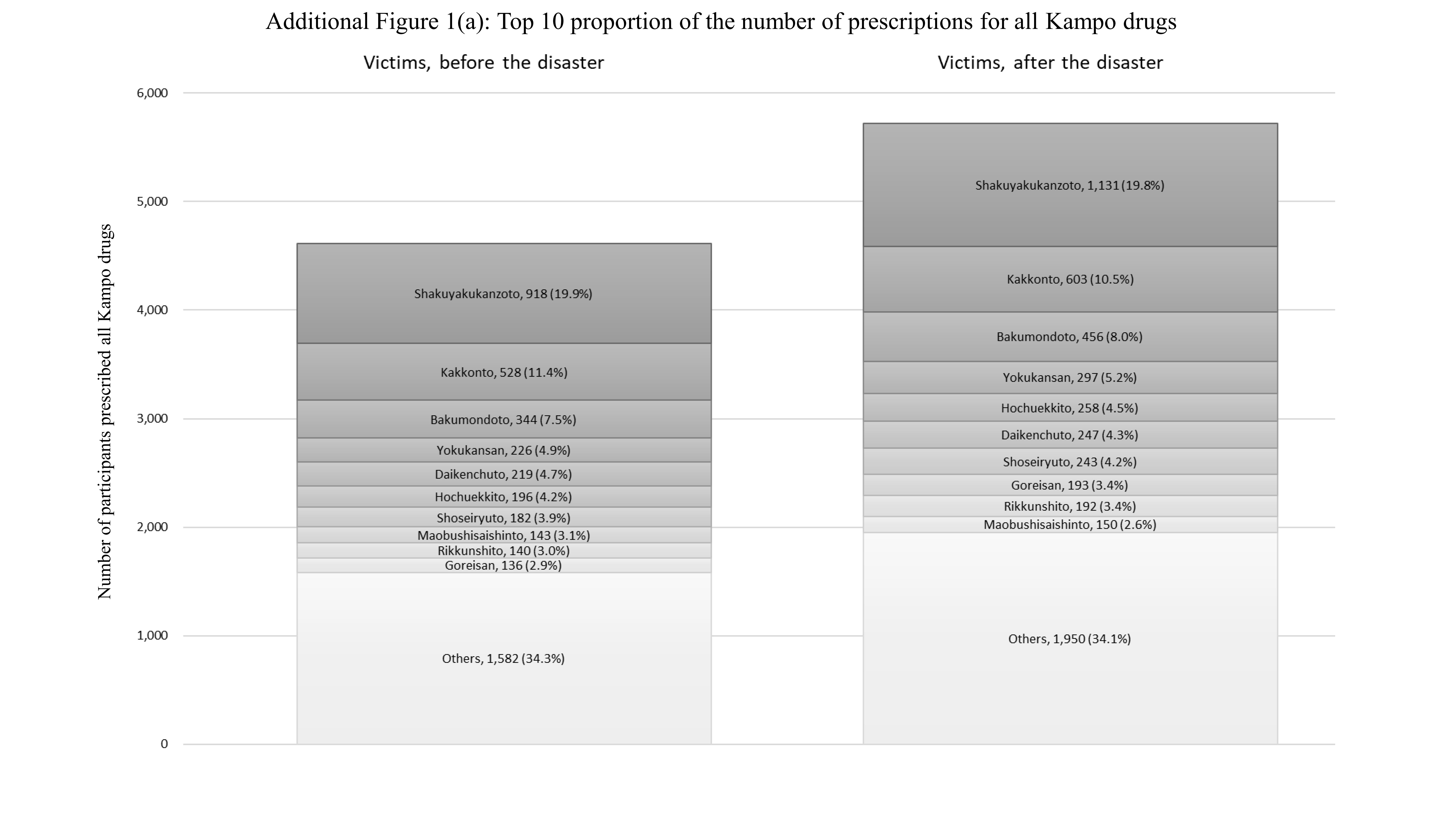

Supplement: Supplementary file 1 [file Image_1.jpg]

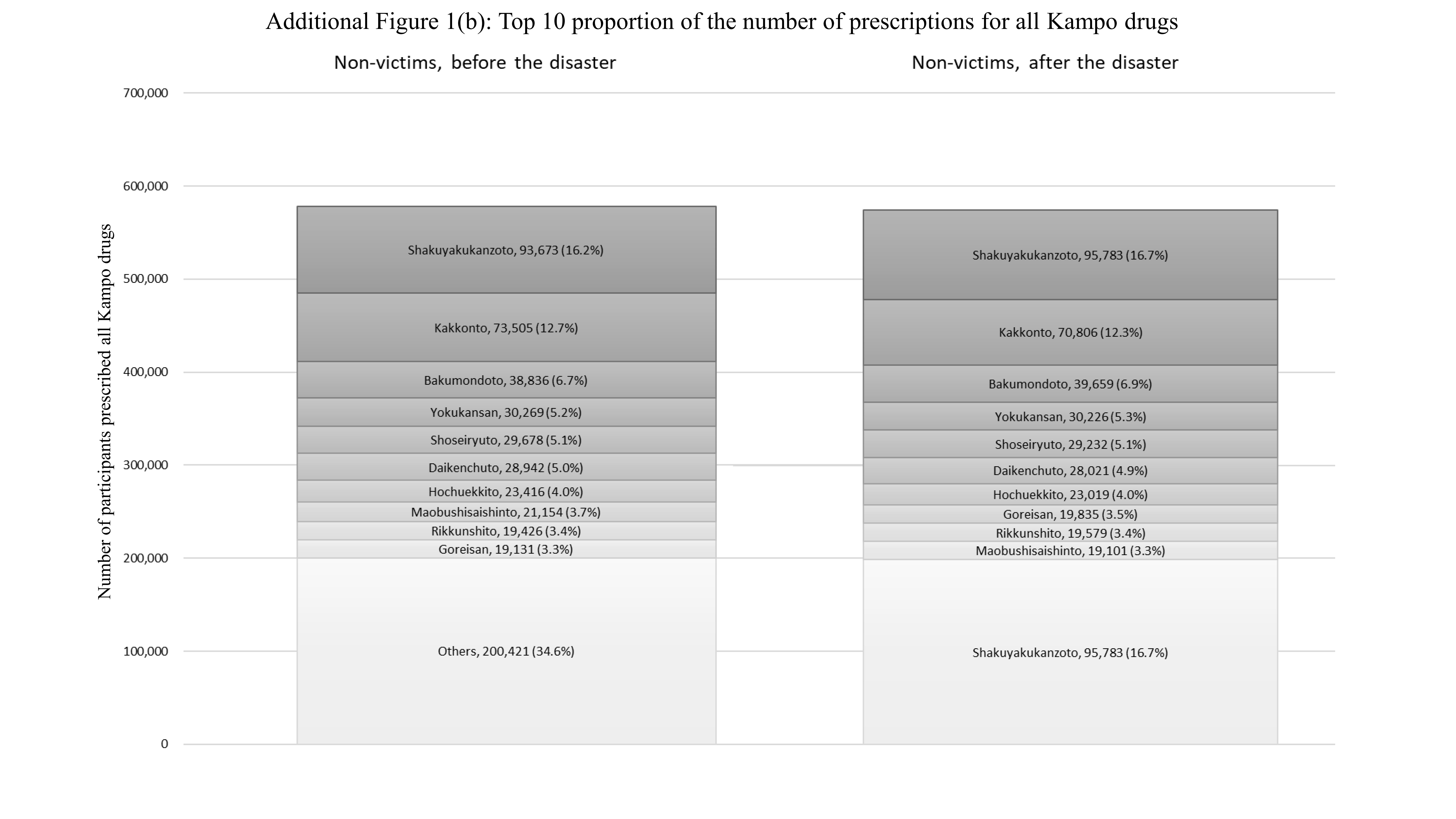

Supplement: Supplementary file 2 [file Image_2.jpg]

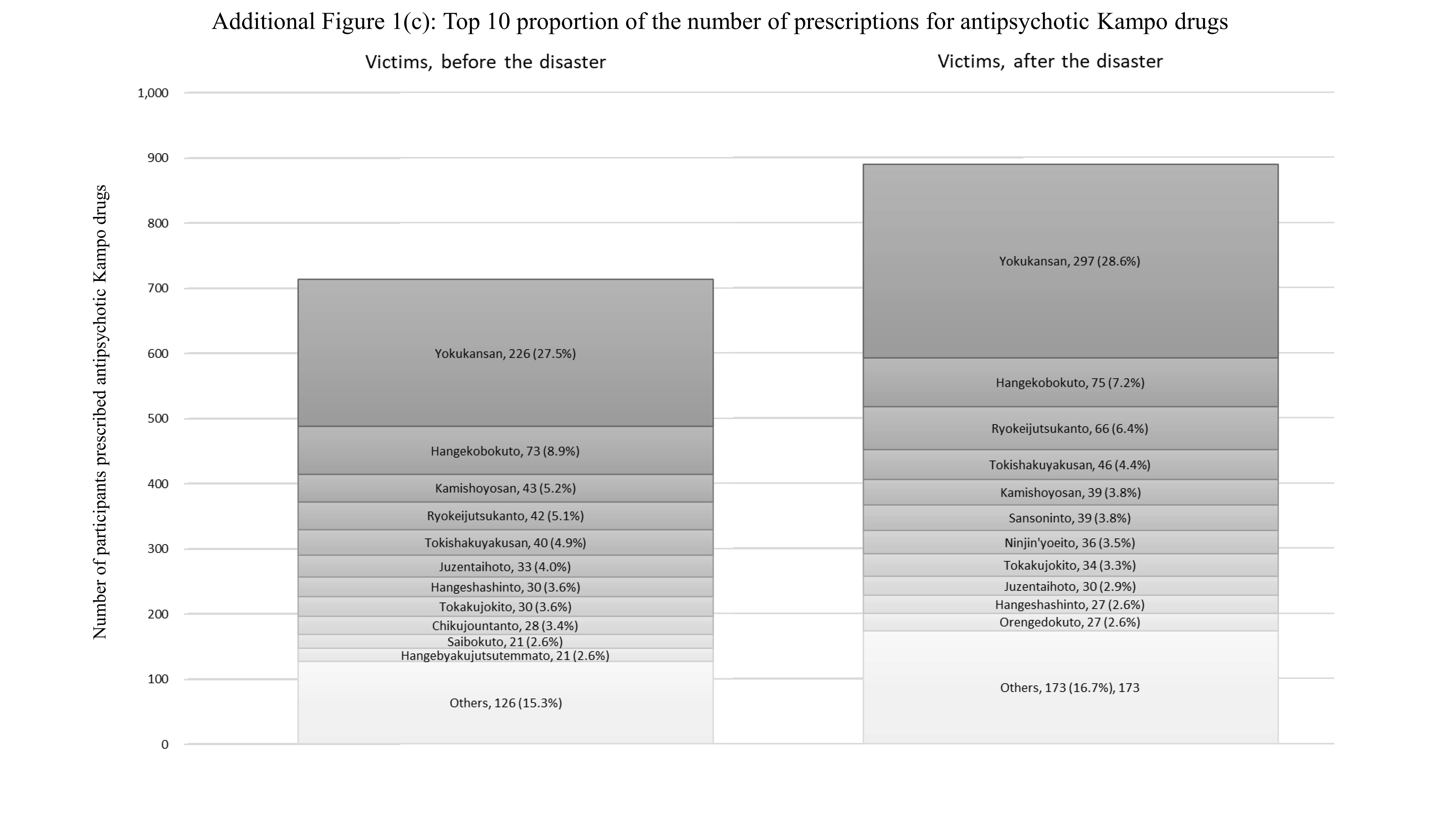

Supplement: Supplementary file 3 [file Image_3.jpg]

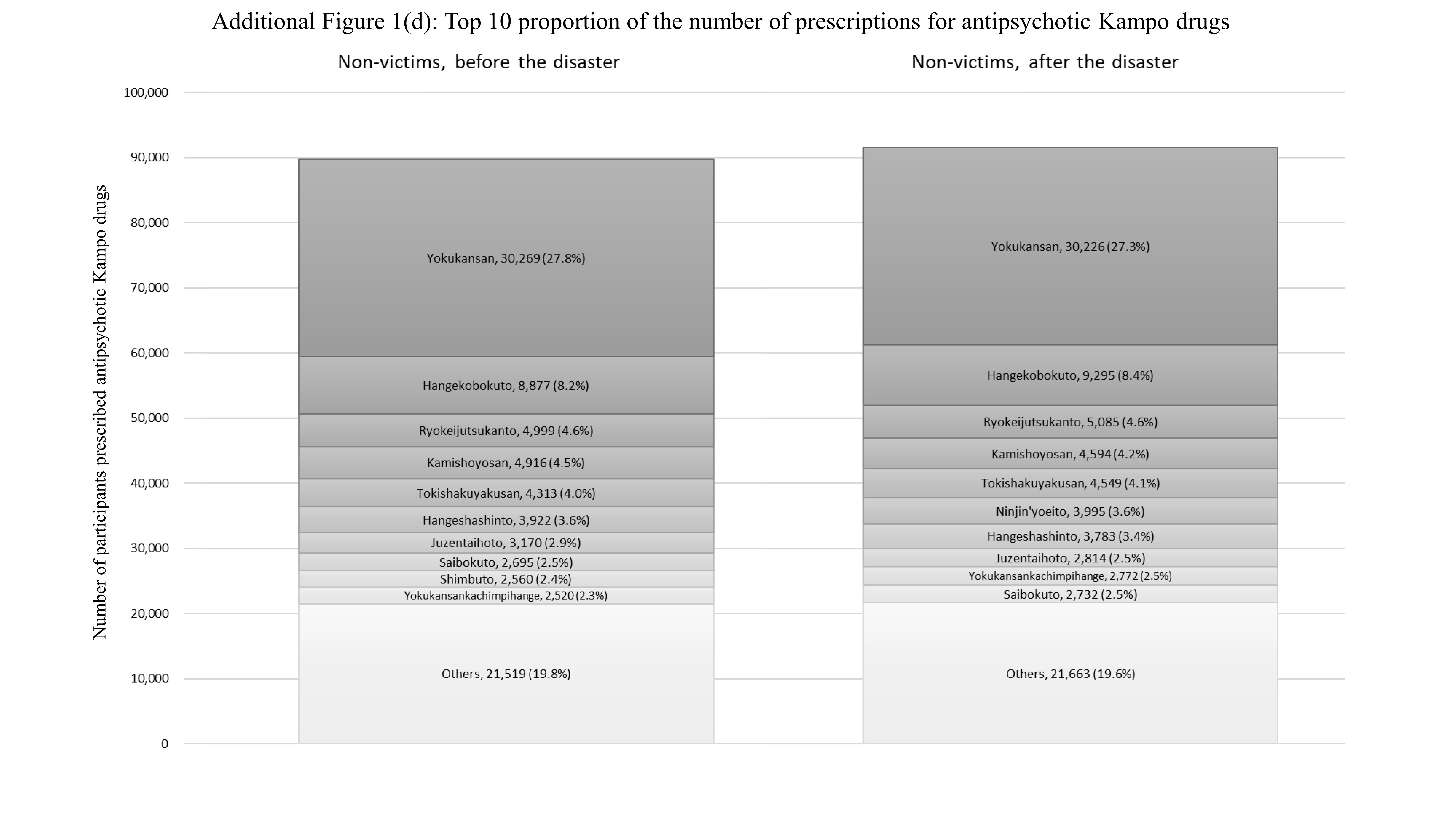

Supplement: Supplementary file 4 [file Image_4.jpg]
